# Haustral loop extraction for CT colonography using geodesics

**DOI:** 10.1007/s11548-016-1497-x

**Published:** 2016-11-16

**Authors:** Yongkai Liu, Chaijie Duan, Jerome Liang, Jing Hu, Hongbing Lu, Mingyue Luo

**Affiliations:** 1 Department of Biomedical Engineering, Tsinghua University, Beijing, 10084 China; 2 Research Center for Biomedical Engineering of Graduate School at Shenzhen, Tsinghua University, Shenzhen, 518055 China; 3 Department of Radiology and Computer Science, State University of New York at Stony Brook, Stony Brook, NY 11794 USA; 4 Department of Gastroenterology, The First Affiliated Hospital of Anhui Medical University, Hefei, Anhui China; 5 Department of Biomedical Engineering, Fourth Military Medical University, Xi’an, 710032 Shanxi China; 6 Department of Radiology, The Sixth Affiliated Hospital of Sun Yat-sen University, Guangzhou, 510630 China

**Keywords:** Virtual colonoscopy, Geodesics, Haustral loop, CTC

## Abstract

**Purpose:**

The human colon has complex geometric structures because of its haustral folds, which are thin flat protrusions on the colon wall. The haustral loop is the curve (approximately triangular in shape) that encircles the highly convex region of the haustral fold, and is regarded as the natural landmark of the colon, intersecting the longitude of the colon in the middle. Haustral loop extraction can assist in reducing the structural complexity of the colon, and the loops can also serve as anatomic markers for computed tomographic colonography (CTC). Moreover, haustral loop sectioning of the colon can help with the performance of precise prone–supine registration.

**Methods:**

We propose an accurate approach of extracting haustral loops for CT virtual colonoscopy based on geodesics. First, the longitudinal geodesic (LG) connecting the start and end points is tracked by the geodesic method and the colon is cut along the LG. Second, key points are extracted from the LG, after which paired points that are used for seeking the potential haustral loops are calculated according to the key points. Next, for each paired point, the shortest distance (geodesic line) between the paired points twice is calculated, namely one on the original surface and the other on the cut surface. Then, the two geodesics are combined to form a potential haustral loop. Finally, erroneous and nonstandard potential loops are removed.

**Results:**

To evaluate the haustral loop extraction algorithm, we first utilized the algorithm to extract the haustral loops. Then, we let the clinicians determine whether the haustral loops were correct and then identify the missing haustral loops. The extraction algorithm successfully detected 91.87% of all of the haustral loops with a very low false positive rate.

**Conclusions:**

We believe that haustral loop extraction may benefit many post-procedures in CTC, such as supine–prone registration, computer-aided diagnosis, and taenia coli extraction.

## Background

**Fig. 1:**
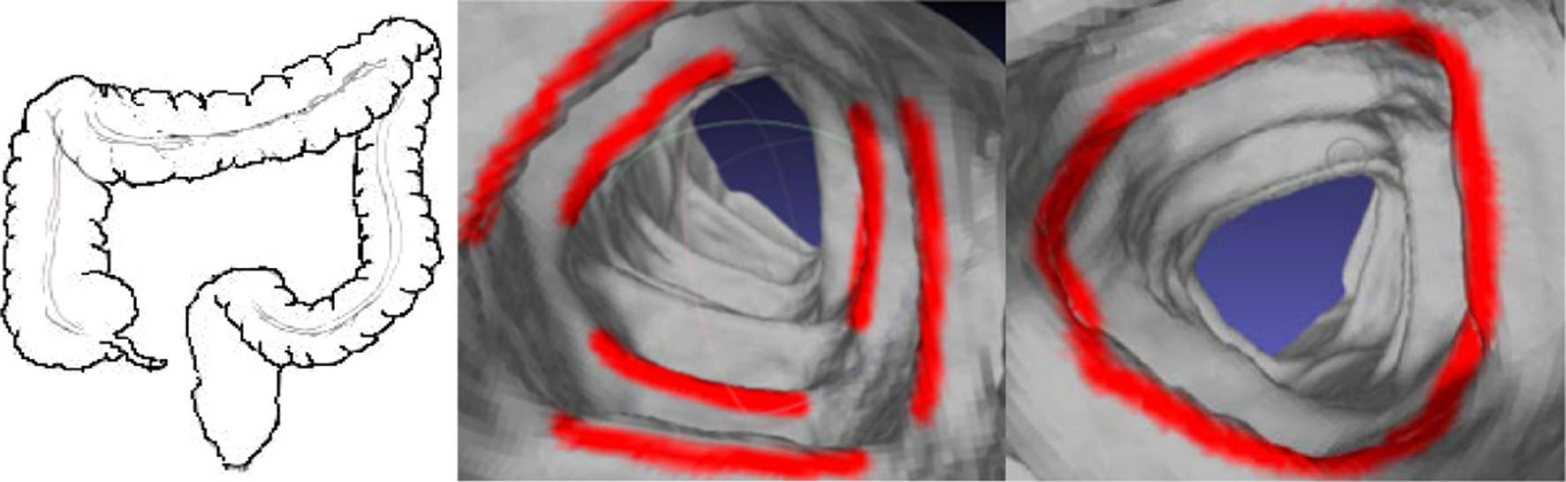
*Left* anatomy of the colon. *Middle* red regions are the haustral folds of the colon. *Right* the three haustral loops are extended into a circular ring

**Fig. 2:**
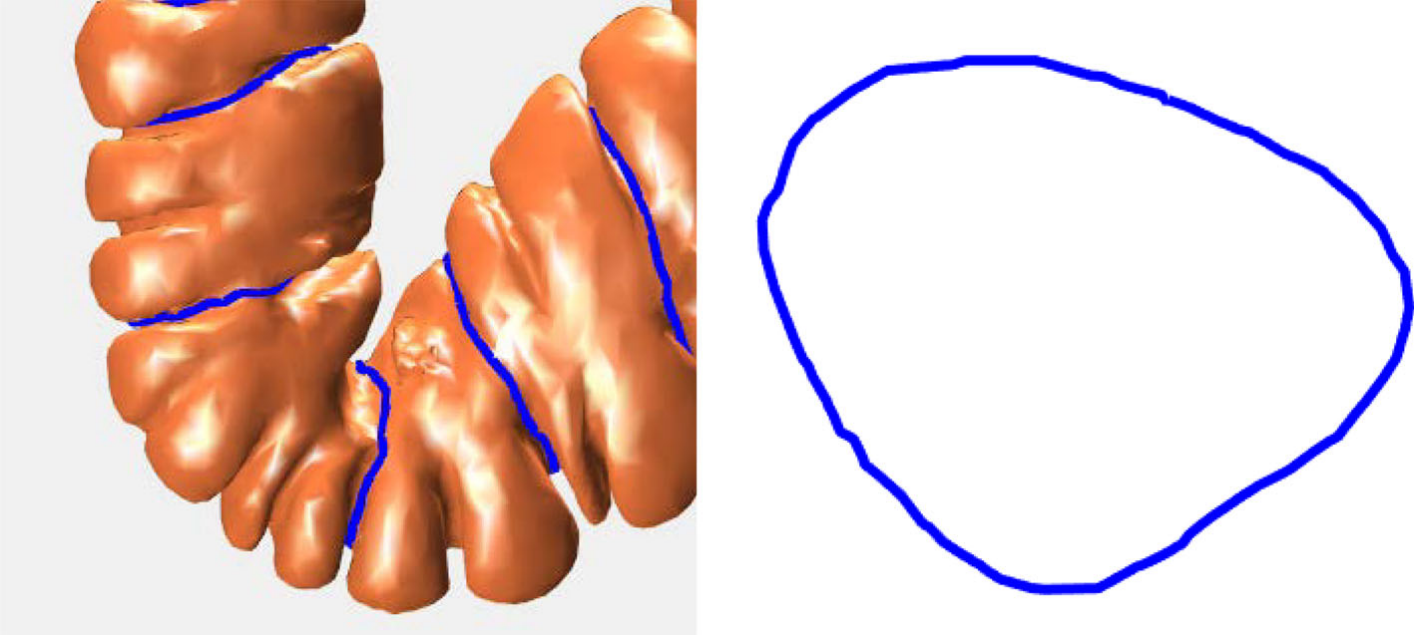
*Left* haustral loops (on the colon); *blue curves* are the haustral loops. *Right* a single haustral loop

Colon cancer is the third most commonly diagnosed cancer in the USA according to recent statistics from the American Cancer Society [1]. It is mostly caused by polyps; thus, effective screening and removal of polyps can greatly reduce the incidence of this disease [2]. Currently, optical colonoscopy is the gold standard for inspecting the entire colon; however, this procedure is time consuming and uncomfortable for the patient and is occasionally associated with serious complications such as colonic perforation [3]. Computed tomographic colonography (CTC) has recently emerged as a reliable, minimally invasive technique for colon cancer screening. This technique reconstructs a three-dimensional (3D) patient-specific colon mucosa model from patient volumetric CTC data and generates a 3D virtual endoscopic layout within the lumen of this model [4, 5], which physicians can use to detect and locate the colonic polyps.

**Fig. 3:**
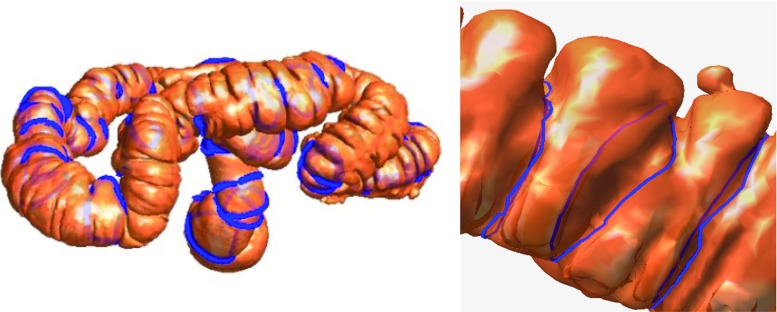
*Left* overview of the haustral loops on the colon surface. *Right* haustral loops in a local region

**Fig. 4:**
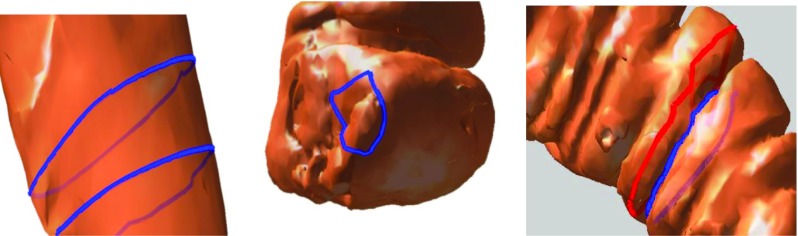
*Left* the loop does not crosscut the surface, so it is not regarded as a haustral loop. *Middle* the loop encircles the smooth area of the surface, so it is not regarded as a haustral loop. *Right* the *blue* and *red lines* both crosscut the colon, but the *red line* is not a haustral loop since the region that it crosscuts is not relatively concave

The surroundings of the colon are extremely complex, as shown in Fig. 1 (left panel), due to the fact that this organ turns and twists in various directions and its folds significantly vary in different locations [6]. The haustral folds represent the three major circumferential folds of mucosa within the colon and are formed by the circumferential contraction of the inner muscular layer, as shown in Fig. 1 (middle panel). If the curves (also known as haustral loops) that highly encircle the concave region of the colon are extracted and used to section the colon, the complexity of this organ would be expected to decrease. This would allow clinicians to focus on one segment at a time instead of the entire colon in all of its complexity.

Aimed toward sectioning the colon crosswise, the haustral loop, which crosscuts the colon, is the curve that encircles the highly concave region of this organ (i.e., three major circumferential folds) and is approximately the shape of triangle, as shown in Fig. 2 (see first part of Methods section for characteristics of the haustral loops).

Haustral loop extraction can also contribute to precise registration of colon surfaces extracted from prone and supine images. Previous works concerning colon registration have utilized information from the centerline of the colon [7–10]. However, these methods only provide $1^{\circ }$ of freedom related to the colonic surface. Although the centerline can represent the local stretching and shrinking along the path of colon, it cannot account for local deformations or twists on its surface. The colon structure is extremely complicated with its large deformations and twists [6], and the colon surface is divided into five segments. Recently, Zeng et al. [11] proposed a method based on conformal mapping combined with feature matching to establish correspondence between the prone and supine surfaces. In Roth et al. [6], the authors provided a one-to-one mapping of the 3D surface to 2D space. These methods can significantly reduce the complexity of the colon, thereby improving registration between the supine and prone colon surfaces [6, 11]. Similarly, using haustral loops to section the colon can cause many segments to be obtained. The entire colon registration work can turn to segment-to-segment registration instead of the entire colon to entire colon registration. This may reduce registration errors caused by the complex geometric structure of the colon.

Extraction of the haustral loops can also aid in the extraction of taenia coli (TC), which anatomically are three separate longitudinal ribbons of smooth muscle in the colon [12, 13]. They are parallel, nearly equidistant, and form a piecewise triple-helix structure from ascending to sigmoid colon segments. TC muscle, with tension, contracts lengthwise to produce the haustral folds, which appear as bulges on the colon wall mucosa. The formation of the haustral loops is also due to the circumferential contraction of the inner muscular layer of the colon, resulting in bulges that are reflected in the angular parts of the approximate triangles. If the positions of the angles in these triangles are extracted, TC extraction can be promoted.

This paper presents an accurate and innovative method for extracting haustral loops for CT virtual colonoscopy. In Methods section, the characteristics of the geodesics are presented followed by the haustral loop extraction algorithm. In Results section, the evaluation design is outlined and the results obtained from clinicians are provided. Then, some results are drawn following some discussions. Finally, experimental conclusions are drawn.

## Methods

### Characteristics of haustral loops

As shown in Fig. 3, the haustral loop encircles the highly concave region of the colon, generally in the approximate shape of a triangle. In addition, the plane through which the haustral loop passes is relatively vertical to the colon. The haustral loop has three main characteristics that help distinguish it from erroneous loops.

#### The loop must crosscut the concave region of the colon

As shown in Fig. 4 (left panel), sometimes the haustral loop is found in a local area on the surface. Although the local concave regions of the colon can generate a loop, this loop is not a haustral loop since it does not crosscut the concave region of the colon. The loop in Fig. 4 (right) panel crosscuts the colon, but encircles the smooth area of the surface, and as such, is not a haustral loop.

#### The loop must encircle the colon on the surface, not inside or outside it

**Fig. 5:**
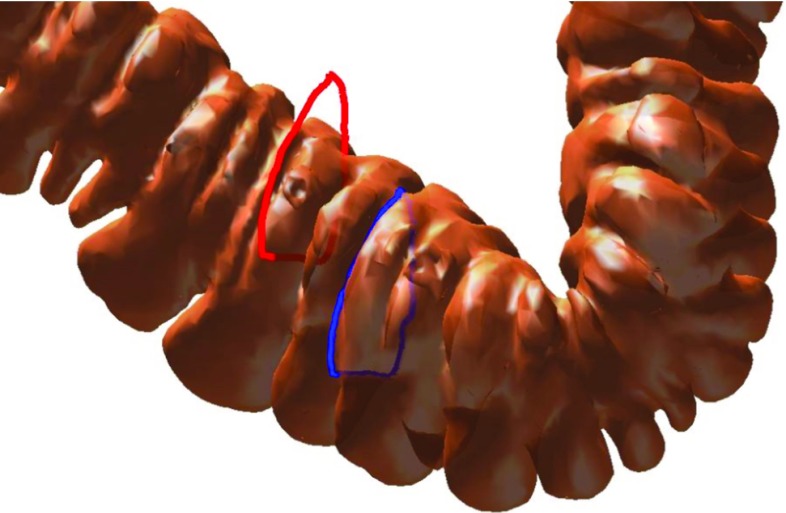
We show two loops on the colon. The *red loop* is partly outside and partly inside the surface. Only a small number of points can exist on the surface, so it is not a haustral loop. The points of the blue loop all lie on the surface, so it is a haustral loop according to the definition

Each point along the loop must appear on the surface of the colon. An illustration is shown in Fig. 5.

#### The plane of the haustral loop is parallel to the cross section of the colon

**Fig. 6:**
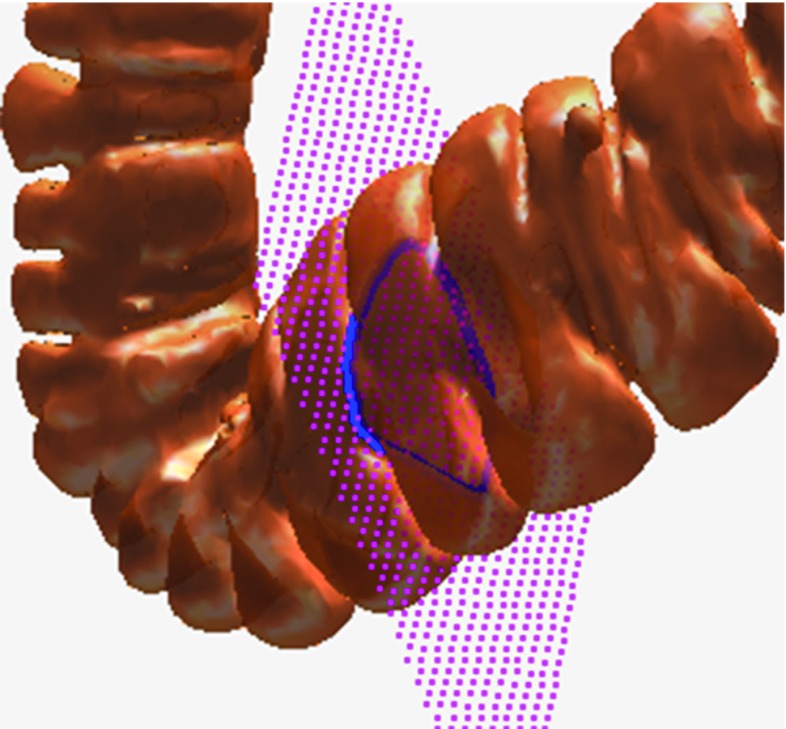
The *blue line* is a haustral loop; the *wine red points* represent the plane of the *blue line*

As shown in Fig. 6, the plane of the haustral loop, which is represented by blue line, is parallel to the cross section (wine red points) of the colon.

### Haustral loop extraction algorithm

**Fig. 7:**
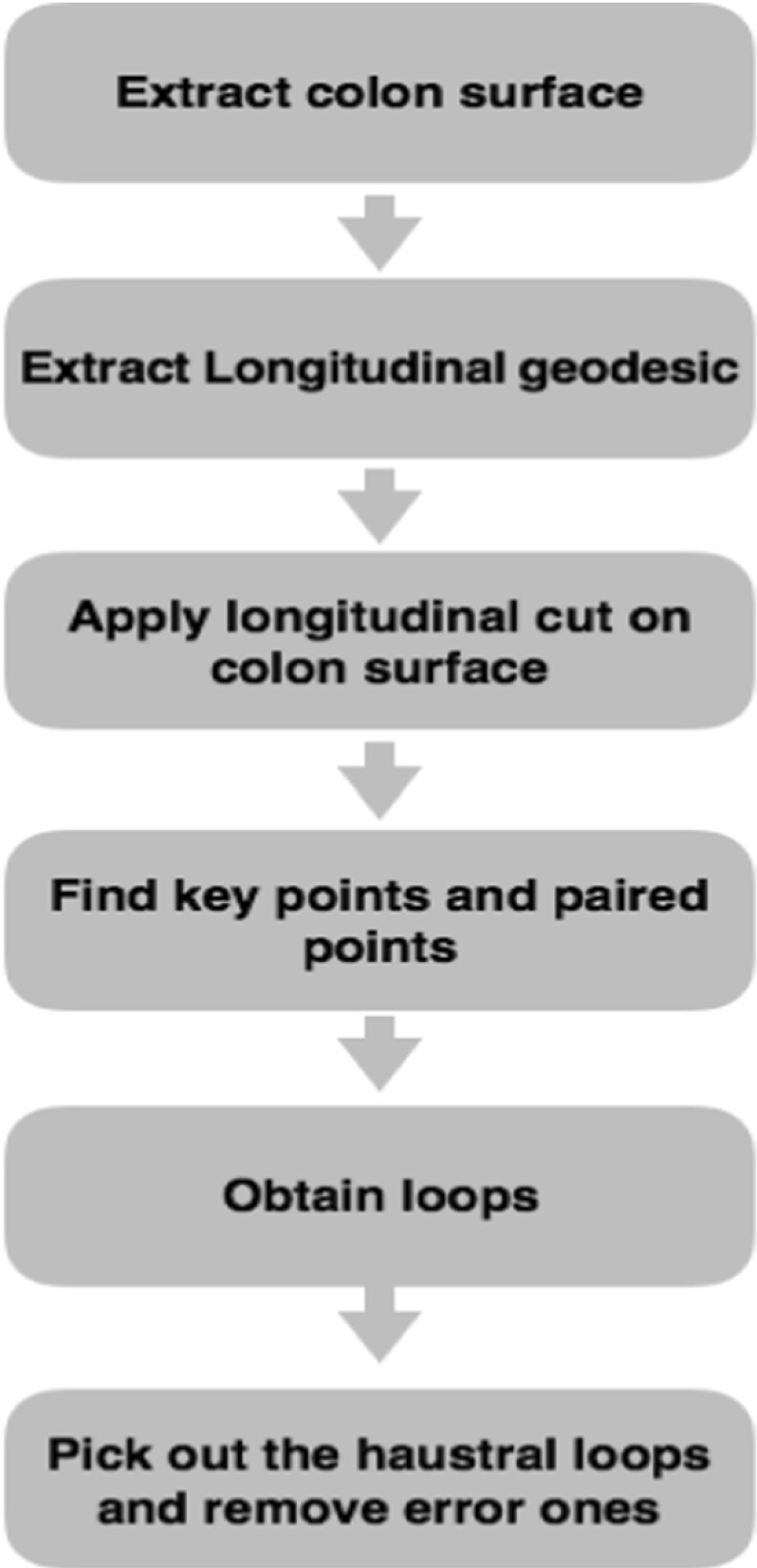
An overview of the proposed haustral loop detection algorithm

An overview of the proposed haustral loop detection algorithm is shown in Fig. 7. We first extracted the colon surface, after which two points were manually selected as start and end points, between which the longitudinal geodesic (LG) was computed. Next, the colon was cut along the LG. It is worth nothing that many paired points used for seeking the loops that encircle the three major circumferential folds are located based on the points on the LG. Next, for each paired point, the shortest distance (geodesic line) between the paired points twice was calculated, namely, one on the original surface and the other on the cut surface. Finally, the two geodesics were combined to form a full loop. Because loops are always present that do not satisfy the standard definition of haustral loops, we devised a detection method to automatically remove these erroneous while identifying actual haustral loops.

#### Extraction of the colon surface

The colon lumen image from the CTC image was first obtained using a previously published method [14, 15]. Then, the level-set method [16] was applied to this image to retrieve a one voxel-layer, representing the colon wall. Finally, the colon surface represented by the triangle mesh S was built using the marching cube method [17].

#### Extraction of LG

**Fig. 8:**
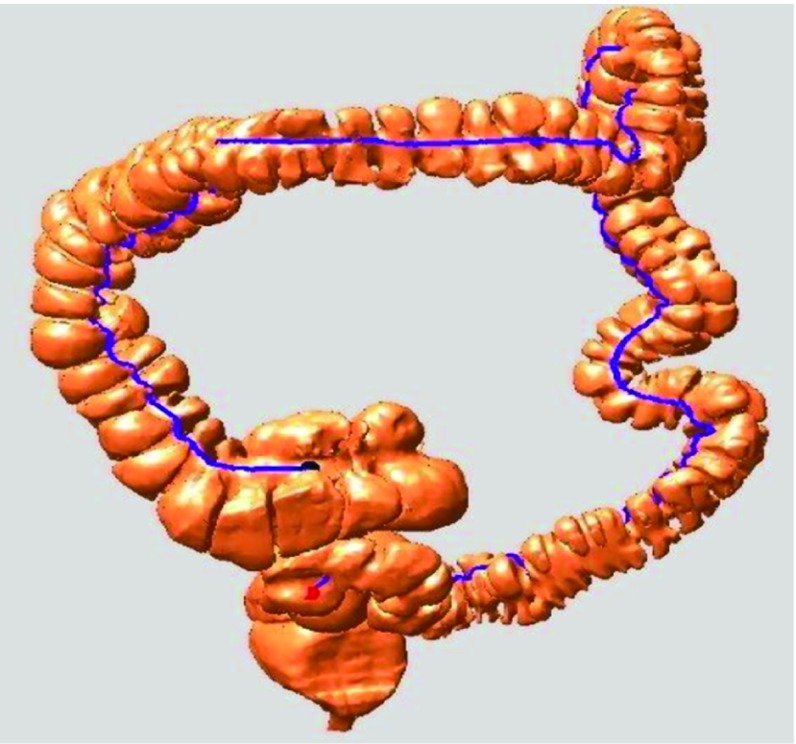
The *blue curve* is the LG connecting the start point (*black point*) and end point (*red point*)

As shown in Fig. 8, we created the LG. After the start and end points were manually specified, we computed by connecting start and end points of the geodesics, which was termed as LG. The purpose of extracting the LG is to apply the longitudinal cut on the colon surface and to seek the key points used to generate paired points.

#### Applying a longitudinal cut to the colon’s surface

**Fig. 9:**
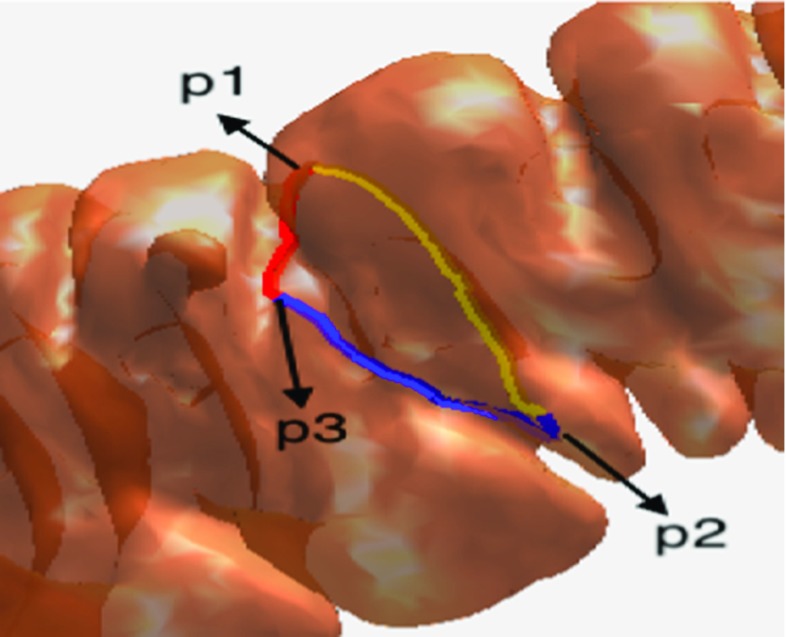
*p1*, *p2*, and *p3* are the points on the three major circumferential folds that were selected manually. The *red line* is the geodesic between p1 and p3, the *yellow line* is the geodesic between p1 and p2, and the *blue line* is the geodesic between p2 and p3

As shown in Fig. 9, to form the haustral loop, three points (p1, p2, p3) should lay on the three major circumferential folds, and the haustral loop should encircle these three folds and highly concave regions. For any two of the three points, we could calculate the geodesic between them. The geodesic between any two points should pass through the highly concave region between the two points since only this route distance between two points is smallest.

**Fig. 10:**
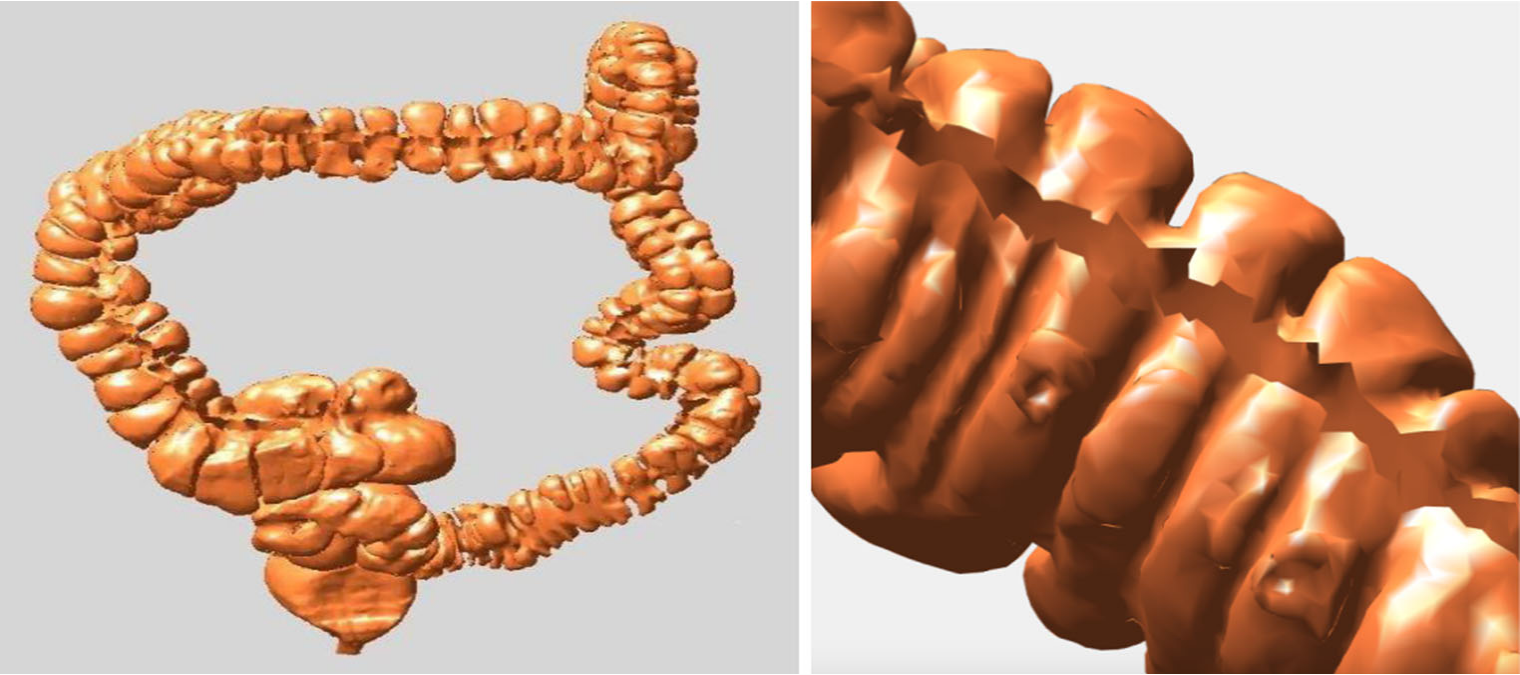
*Left* overview of the cut surface. *Right* local region of the cut surface

By connecting the three geodesics, a complete haustral loop passing through the three major circumferential folds could eventually be obtained. This is similar to the principal that at least three satellites are needed to encompass the entire surface of the earth. If we only selected two points manually, we could not obtain a complete loop. For example, if the two points were p1 and p2, we could only obtain the yellow line and not the complete loop, as shown in Fig. 9. However, manually selecting the points is tedious work. For each haustral loop, three adjacent points on the highly concave region on the three circumferential folds should be selected, and the manual points directly influence the extraction of the haustral loop. To solve this problem, we applied a longitudinal cut. As illustrated in Fig. 10, the vertexes on the colon surface, with distances to any point in LG that were less than threshold, were removed. The rest colon surface was called the cut surface (CS).

**Fig. 11:**
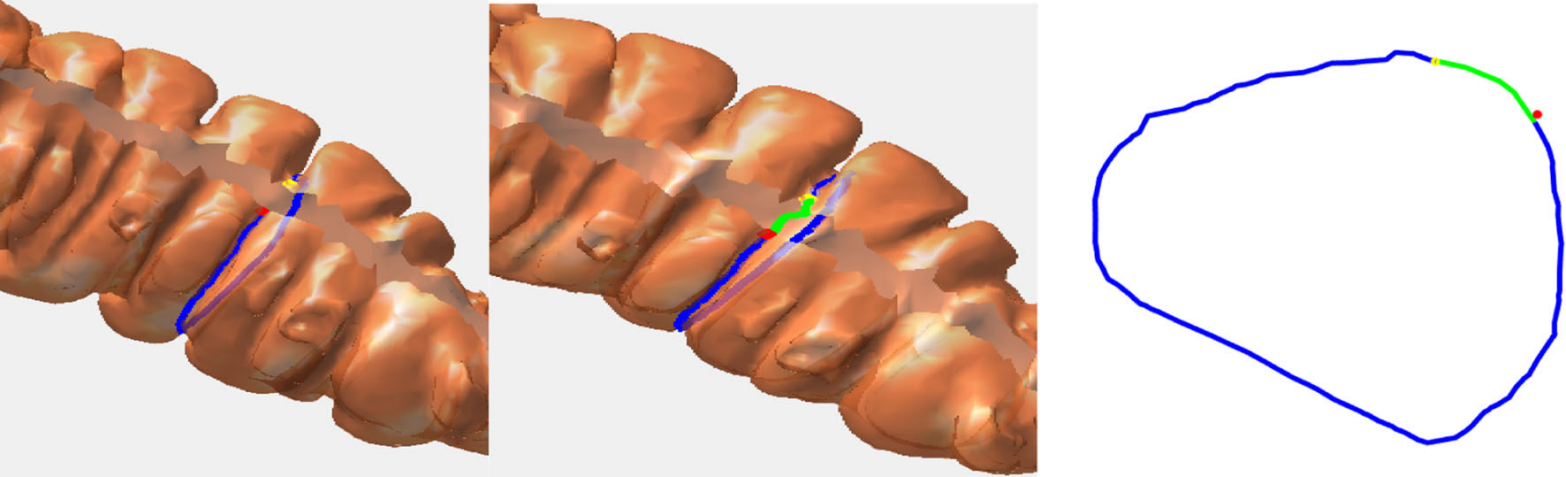
*Left* *yellow* and *red points* are paired points and the *blue line* is the geodesic connecting the two points on the cut colon surface. *Middle* *green line* is the geodesic connecting the two points on the original colon surface. *Blue line* is the long geodesic and the *green line* is the short geodesic. The *blue* and *green lines* together form a loop. *Right* The potential haustral loop

When we applied a cut, as shown in Fig. 11, we only needed to specify two points, between which we computed the geodesics twice, one on the entire colon surface and the other on the cut surface. The two points were called paired points. Compared to the cut surface, this cut was much smaller, so the path length of the geodesic connecting the paired points on the whole colon surface was much smaller than that of the geodesic connecting the paired points on the cut surface. The geodesic on the whole colon surface is called short geodesic, and the other geodesic is called long geodesic. Obviously, connecting the long and short geodesics can form a potential haustral loop. The paired points were on both sides of the cut, which we referred to as one-side point and another-side point.

**Fig. 12:**
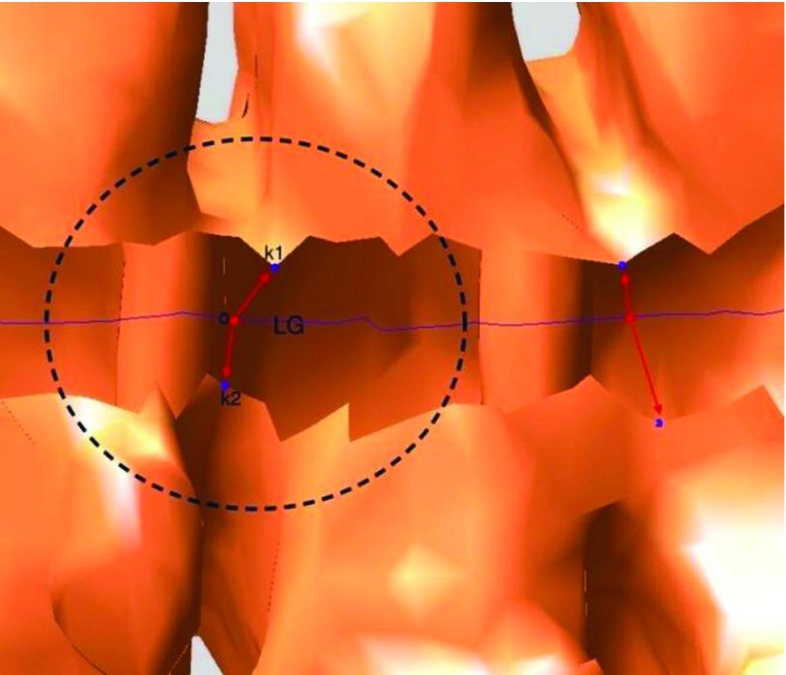
The points *k1* and *k2* are one-side point and other-side point. The *blue line* is the LG. Point O was selected from the LG at the interval

#### Find key and paired points

We were able to utilize the LG information to obtain the paired points. As shown in Fig. 12, some points along the LG of the colon at intervals were roughly extracted and regarded as key points. For each key point, we first found the point on the CS whose distance to the key point was smallest among all of the points on the CS; this point was called the one-side point and was on one side of the cut. Then, angles between the vector connecting the key point and the one-side point and the vectors connecting the key point and the other points on the CS were calculated. The other points whose corresponding angles were greater than $\pi /2$ were regarded as candidate points for seeking the other-side point. The point in the candidate points whose distance to the key point was the smallest among all of the points in the candidate points was the other-side point.

#### Obtain the loops

**Fig. 13:**
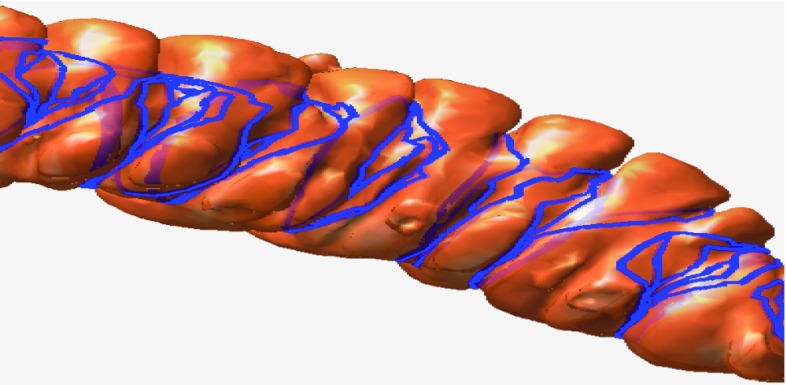
Many loops between the paired points are generated

We obtained a pair of paired points based on a key point. Then, the geodesic between the paired points on the CS could be calculated, and the geodesic was called the long geodesic. The geodesic between the paired points on the original colon surface was also calculated and called the short geodesic. A loop was obtained by connecting the short and long geodesics. Each pair of paired points could generate one loop. In the end, we obtained many loops since there were many selected key points, as shown in Fig. 13.

#### Identifying haustral loops and removing erroneous ones

**Fig. 14:**
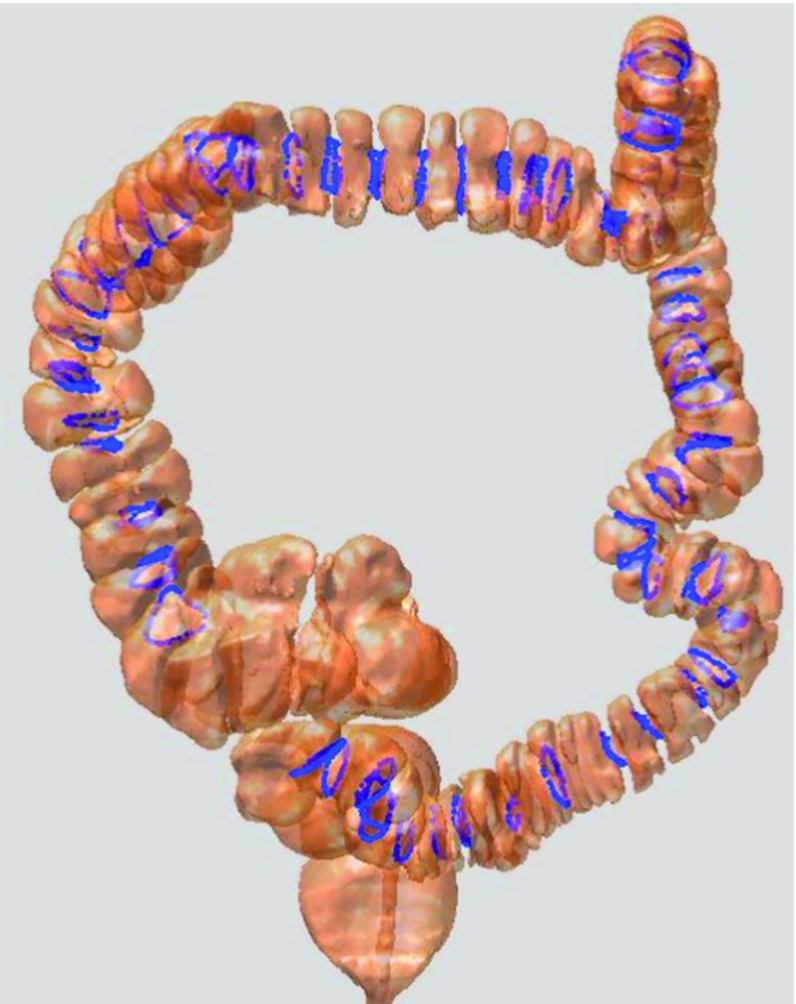
The *blue lines* are the haustral loops displayed on the surface

To identify the haustral loops, we designed the following algorithm. Loops that were too close to each other (distance between centroids of haustral loops) always encircled the same concave region of the colon; these loops were called adjacent loops. The path length of the loop was exploited to identify the haustral loop among adjacent loops. Based on these characteristics, the haustral loop must always encircle highly concave regions, so the path length must be smallest among all of the adjacent loops. In addition, the haustral loop must be composed of a certain number of points. Based on our experiments, those loops with too few points were always the ones that appeared on the surface, so we removed them. Figure 14 shows the haustral loops displayed on the colon’s surface.

## Results and discussion

### Data preparation

The proposed methodology was applied to 10 CTC datasets of patient collected after informed consent. Their colon cleansings were performed with standard pre-colonoscopy or barium enema bowel preparation with oral fecal tagging. Each patient was scanned in both the supine and prone positions by 4- and 8-MDCT scanners (Light Speed Ultra, GE Medical Systems, Milwaukee, WI) resulting in 10 CT scans. We performed electronic colon cleansing incorporating the partial volume effect [18]. The CT images were segmented using a MAP-EM algorithm [15] for both colon lumen cleansing and mucosa layer extraction. The extracted mucosa layer was shrunk by a level-set method for a single voxel-thick surface [19]. The marching cube method was used to reconstruct a mesh model for the inner wall or mucosa surface. The reconstructed meshes have many sprue handles. However, all of the fake handles were removed via topological de-noising. Although the size and resolution of each CT volume varies from dataset to dataset, the general data size is approximately $512 \times 512 \times 450$ voxels, and the general resolution is approximately $0.7 \times 0.7 \times 1.0$ mm. In this paper, the colon surface was modeled as a topological cylinder and discretely represented by a triangular mesh.

### Results and discussion

The algorithm that we devised allowed haustral loops in the colon to be obtained and allowed clinicians to identify them. The number of the missing and the number of the incorrect haustral loops identified are obtained respectively. The results of the haustral loops are presented in Table 1.

**Table 1:** The results of the haustral loop

Datasets	Number of the haustral loops recognized by algorithm	Number of the incorrect haustral loops identified by clinician	Number of the missing haustral fold loops identified by clinician	Number of the haustral loops identified by clinicians
1	62	1	3	64
2	64	0	6	70
3	56	2	7	61
4	51	2	8	57
5	55	2	10	63
6	52	2	6	56
7	77	1	2	78
8	68	3	5	70
9	49	1	2	50
10	60	2	4	62
Total	594	16	53	631

In Table 1, columns 1 through 5 indicate the serial number of CTC datasets of patient, the number of the haustral loops recognized by algorithm, number of the incorrect haustral loops in identified by clinician, number of the missing haustral fold loops identified by clinician, and the number of the haustral loops identified by clinicians.

Through calculations using the data presented in the chart above, the true positive rate of haustral loops detected by the algorithm was 91.87%, the false positive rate was 2.53%. However, some folds on the colon were not very salient, and adjacent folds that were not circumferential were very close to these less salient folds, making the loop fully encircle the three major circumferential folds.

**Fig. 15:**
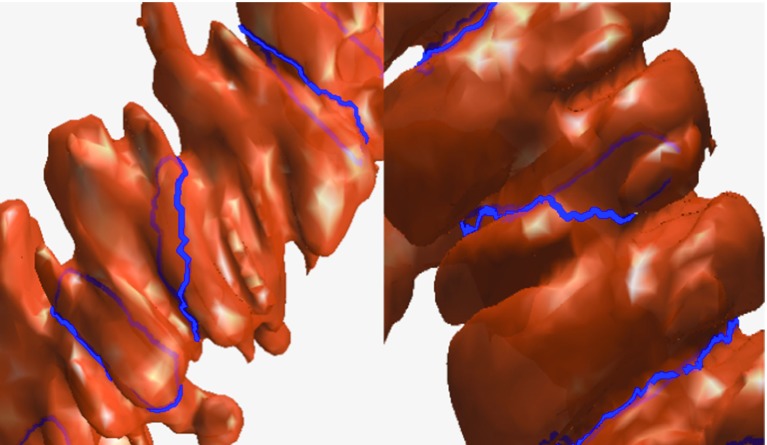
The incorrect haustral loops are displayed on the colon surface

**Fig. 16:**
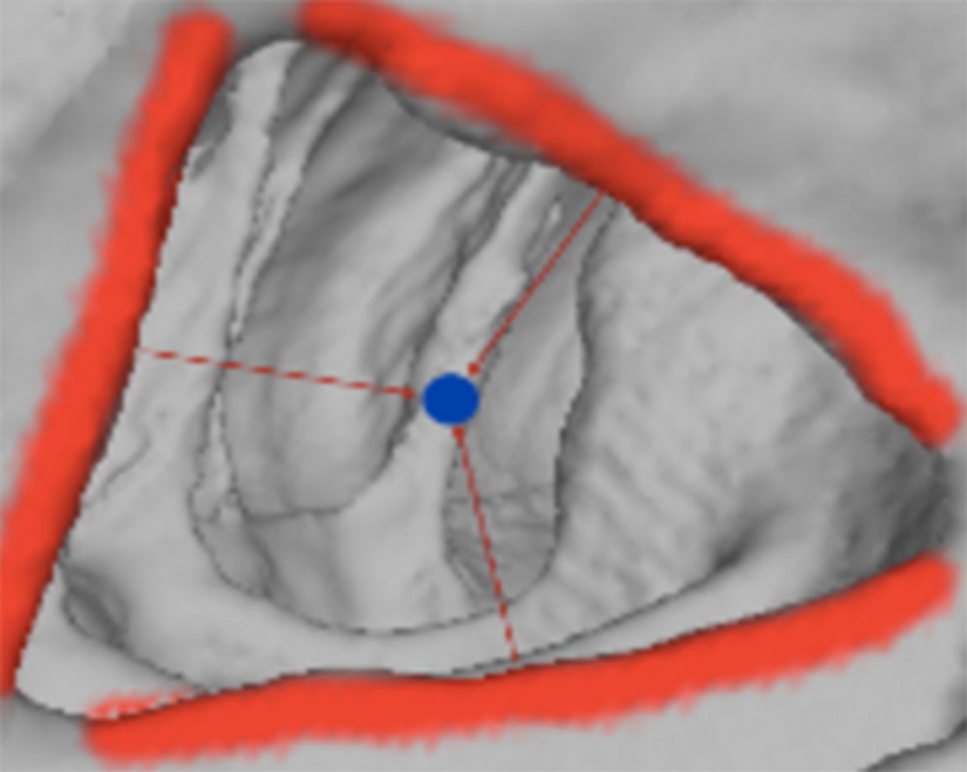
The *red regions* are the grouped haustral folds

The middle blue curve in Fig. 15 is not the correct haustral loop. The line does not encircle the three major circumferential folds and is not parallel to the cross section of the colon, so it is not a haustral loop. To solve this problem, in the future, we will make use of the centerline. The standard haustral loops are generally approximately vertical to the centerline, while angles between these kinds of incorrect loops and the centerline may be much less than $\pi /2$. After we identified the incorrect loops, haustral folds around the incorrect loops were extracted and then the haustral folds were grouped according to whether they composed the three major circumferential folds, as shown in Fig. 16. In the end, geodesics were calculated on these grouped haustral folds.

Regarding the missing haustral loops, here is one of the most common cases.

**Fig. 17:**
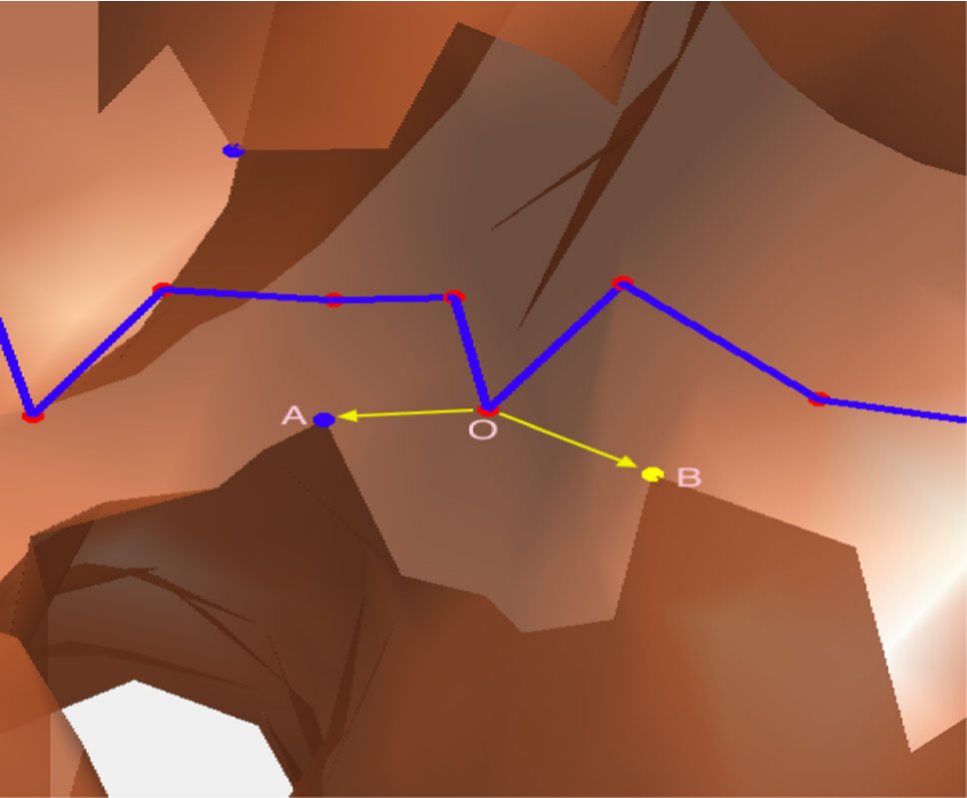
The *red regions* are the grouped haustral folds

As shown in Fig. 17, the red points are the key points on the LG. In our algorithm, for each key point, two paired points were calculated which were expected to be on the two different sides of the incision. Then, a loop can be generated by connecting the two points. However, a key point like O is too close to the border of incision, meanwhile the incision border is not smooth, so angle between vector $\overrightarrow{OA}$ and $\overrightarrow{OB}$ vector is greater than $\pi /2$. These two points (point A, point B) are regarded as paired points. Because they are actually on the same side, they cannot be used to generate a candidate loop, which would lead to a missing haustral loop. Actually, when the key point is closer to the middle of the incision, there is less of a possibility that the paired points are on the same side. In the future, we will smooth the LG before using it to extract key points, which will allow key points to be closer to the middle. In addition, future studies will test the algorithm in more patients, thereby showing its robustness in various clinical conditions.

## Conclusions

The proposed method reduces the complexity of the colon surface though sectioning of the colon. According to the experiments, the algorithm for extracting haustral loops can divide the colon into dozens of segments with high accuracy and specificity. Thus, this method has the potential to contribute to the improvement in registration between the supine and prone colon.
